# Supplementary material for: Warmth Centrality in Social Cognitive Networks of Fairness Reputation Across Players in the Ultimatum and Dictator Games
Source: Behav Sci (Basel). 2025 Nov 11;15(11):1537. doi: 10.3390/bs15111537 (PMC12649631; doi:10.3390/bs15111537)
Supplement: Supplementary file 1 [file behavsci-15-01537-s001.zip › behavsci-3882873-supplementary.pdf]

Supplementary Material

1 Participant

1.1 Participant screening

To ensure the reliability and accuracy of the results, we conducted a thorough data examination. We excluded four participants with incorrect answers to the lie-detection item, four with survey completion times exceeding three standard deviations from the mean, and twelve with average estimates for role distribution per trial exceeded ± 3 standard deviations, suggesting they may have misunderstood the fairness reputation of the roles.

Participants were categorized into distinct SVO basing on the SVO angle derived from the SVO Slider Measure (Murphy et al., 2011). The resulting SVO Slider angles can be easily transformed into corresponding categories as follows: participants with SVO angles between 22.45° and 57.15° were identified as prosocial (n = 89), while participants with SVO angles between -12.04° and 22.45° were identified as individualist (n = 59). This study did not identify any participants with angles indicating altruistic or competitive types. To balance the numbers of the prosocial and individualist, and given the substantial imbalance in group sizes (89 vs. 59), we balanced the two groups to ensure comparable statistical power in the ANOVA. Specifically, we retained 63 prosocial participants with the largest SVO angles, representing stronger prosocial tendencies, and included all individualistic participants (n = 59). Participants whose SVO angles fell on the boundary between groups were all retained to avoid arbitrary exclusion.

As a result, 122 valid participants (61 males, Mage = 20.68 years, SE = 1.94, range = 18-29 years) were included in the final analyses. All exclusions and balancing procedures were determined a priori to ensure data integrity and fair comparison between SVO categories. No participants were excluded due to failed comprehension checks or incomplete data.

1.2 Sampling / power

To detect the main effect of between-subject variables, we chose the "F-tests ANOVA: Repeated measures, between factors" model in G*Power. Assuming a power of 1-β = .90, alpha of

α = .05, effect size of f = .25, number of groups = 2, number of measurements = 4, and correlation among repeated measures of .5 (as dependent variables are highly correlated in different offer levels), the optimal sample size would be 108. To detect the main effect of within-subject variables, we chose the "F-tests ANOVA: Repeated measures, within factors" model in G*Power. Assuming a power of 1-β = .90, alpha of α = .05, effect size of f = .25, number of groups = 2, number of measurements = 4, correlation among repeated measures of .5 (as dependent variables are highly correlated in different offer levels), and nonsphericity correction ε = 1 (default value), the optimal sample size would be 30. To detect the interaction of within- and between-subject variables, we chose the "F-tests ANOVA: Repeated measures, within-between interaction" model. Assuming a power of 1-β = .90, alpha of α = .05, effect size of f = .25, number of groups = 2, number of measurements = 4, correlation among repeated measures of .5 (as dependent variables are highly correlated in different offer levels), and nonsphericity correction ε = 1 (default value), the optimal sample size would be 30. Therefore, to detect both of the main effects and interaction, we chose the largest sample size of 108.

2 Details of survey process

2.1 Comprehension check questions on rule understanding

For the Ultimatum Game, the check question was: "Suppose you are playing as the responder and the proposer distributes ¥10 between you and him. The proposer decides to allocate ¥4 to you and ¥6 to himself. If you accept the offer, how much will each of you receive? If you reject the offer, how much will each of you receive?"; For the Dictator Game, the check question was: "Suppose you are playing as the dictator and can distribute ¥10 between yourself and the recipient. You decide to keep ¥6 for yourself and allocate ¥4 to the recipient. how much will each of you receive? Can the recipient choose to reject?". Participants could proceed only if they answered the check questions correctly; otherwise, they were prompted to review the rules of the decision-making task.

2.2 Decision-making as players

Participants engaged in decision-making processes as player of the corresponding roles, with the aim of enhancing their comprehension of the established rules. As proposers, participants had to decide “How much will you allocate to the responder?”, with the options ranging from 0 to ¥10. As responders, the question was “The proposer offered you ¥1, and keeping ¥9.”, participants had to decide whether to accept or reject the offer. As dictators, participants had to decide “How much will you allocate to the recipient?”, with the options ranging from 0 to ¥10.

2.3 Comprehension check questions on fairness reputations learning

For proposers and dictators, the question was, “On average, how much did this player give to the responder/recipient per trial?” For responders, the question was, “Assuming the proposer distributed ¥2 to the responder and ¥8 to himself in the 11th round, do you think the responder would choose to accept or reject?”.

3 Supplementary results of social cognition of roles under varying fairness reputation conditions

3.1 Fairness reputations manipulation check

For the proposer role, the paired-samples t-test was significant, where $t(121) = 34.00$, $p < .001$. Participants’ estimated mean offer for fair proposers ($M = 4.00 \pm .45$) was significantly higher than for unfair proposers ($M = 2.18 \pm .49$) (Figure. 1A). Furthermore, participants’ estimated mean offers for both fair and unfair proposers closely matched the actual values set in the survey ($M = 3.90$ for fair proposers and $M = 2.10$ for unfair proposers), suggesting the successful manipulation of proposers’ fairness reputations in this study.

For the responder role, the chi-square test was significant, where $\chi^2 = 164.11$, $p < .001$. For fair responders, only 9 participants (7.38% of the total sample) believed that the responder would

choose to accept an unfair offer in the 11th trial. For unfair responders, 109 participants (89.34% of the total sample) believed that the responder would choose to accept an unfair offer in the 11th trial, suggesting the successful manipulation of responder's fairness reputations in this study (Figure. 1B).

For the dictator role, the paired-samples t-test was significant, where $t(121) = 29.15, p < .001$. Participants' estimated mean offer for fair dictators ($M = 4.06 \pm .41$) was significantly higher than for unfair dictators ($M = 2.23 \pm .67$) (Figure. 1C). Furthermore, participants' estimated mean offer for both fair and unfair closely matched the actual values set in the survey ($M = 3.90$ for fair dictators and $M = 2.10$ for unfair dictators), suggesting the successful manipulation of dictators' fairness reputations in this study.

3.2 Homogeneity test of variance on social cognition of proposers and dictators

For the ANOVA on warmth ratings, the Box's M test revealed significant differences in group covariance matrices, where Box's M = 30.40, $F(2, 93) = 2.93, p = .001$. For prosocial participants: the Levene's test on the variance of warmth ratings for proposers was significant, where $F(1, 124) = 11.65, p = .001$, indicating that the variance of warmth ratings for fair proposers ($S^2 = 1.23$) was greater than that for unfair proposers ($S^2 = .49$); the Levene's test on the variance of warmth ratings for dictators was significant, where $F(1, 124) = 33.12, p < .001$, indicating that the variance of warmth ratings for fair dictators ($S^2 = 1.67$) was greater than that of unfair dictators ($S^2 = .47$). For individualistic participants: the Levene's test on the variance of warmth ratings for proposers was significant, where $F(1, 116) = 36.24, p < .001$, indicating that the variance in warmth ratings of fair proposers ($S^2 = 1.85$) was greater than that of unfair proposers ($S^2 = .33$); the Levene's test on the variance of warmth ratings for dictators was significant, where $F(1, 116) = 4.45, p = .001$, indicating that the variance in warmth ratings of fair dictators ($S^2 = 1.85$) was greater than that of unfair dictators ($S^2 = 1.28$) (Figure. S1).

Box's M test assessed no significant differences in group covariance matrices of other ratings (fairness, trustworthiness, altruism, cooperation, or competence).

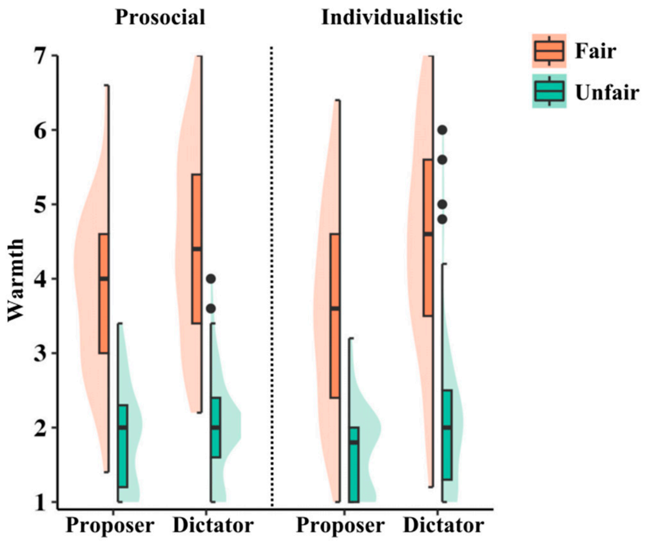

Figure S1. For both prosocials and individualists, the variances of warmth ratings are greater for fair proposers and fair dictators with a wider distribution, than for unfair proposers and dictators with a narrower distribution.

3.3 ANOVA results on social cognition of proposers and dictators

The ANOVA was conducted with fairness ratings as the dependent variable. We found a significant main effect of the fairness reputation, where $F(1, 120) = 686.15$, $p < .001$, $\eta_p^2 = .85$, 95% CI [.67, .79]. The post hoc analysis indicated that fair players ($M = 4.35 \pm .10$) were perceived as fairer than unfair players ($M = 1.87 \pm .06$). The main effect of role was also significant, where $F(1, 120) = 54.79$, $p < .001$, $\eta_p^2 = .31$, 95% CI [.10, .27]. The post hoc analysis indicated that proposers ($M = 2.83 \pm .07$) were perceived as less fair than dictators ($M = 3.38 \pm .08$). The interaction between role and SVO was significant, where $F(1, 120) = 9.06$, $p = .003$, $\eta_p^2 = .07$, 95% CI [.00, .09]. The simple effect analysis indicated that for prosocial participants, proposers ($M = 2.93 \pm .10$) were perceived as less fair than dictators ($M = 3.25 \pm .11$), $p = .002$; and for individualistic participants, proposers ($M = 2.74 \pm .18$) were also perceived as less fair than dictators ($M = 3.51 \pm .12$), $p < .001$, with a larger difference than that for prosocial participants. No other main effects or interactions were significant.

The ANOVA was conducted with trustworthiness rating as the dependent variable. We found a significant main effect of fairness reputation, where $F(1, 120) = 393.18$, $p < .001$, $\eta_p^2 = .77$, 95% CI [.53, .68]. The post hoc analysis indicated that fair players ($M = 4.27 \pm .10$) were perceived as more trustworthy than unfair players ($M = 2.30 \pm .08$). The main effect of role was significant, where $F(1, 120) = 10.51$, $p < .002$, $\eta_p^2 = .08$, 95% CI [.00, .10]. The post hoc analysis indicated that proposers ($M = 3.12 \pm .09$) were perceived as less trustworthy than dictators ($M = 3.45 \pm .09$). The interaction between fairness reputation and role was significant, where $F(1, 120) = 14.29$, $p < .001$, $\eta_p^2 = .11$, 95% CI [.01, .12]. The simple effect analysis indicated that fair proposers ($M_{FP} = 3.94 \pm .12$) were perceived as more trustworthy than unfair proposers ($M_{UP} = 2.30 \pm .11$), $p < .001$; fair dictators ($M_{FD} = 4.61 \pm .12$) were also perceived as more trustworthy than unfair dictators ($M_{UD} = 2.29 \pm .10$), $p < .001$, with a larger difference than that for proposers. No other main effects or interactions were significant.

The ANOVA was conducted with altruism ratings as the dependent variable. We found a significant main effect of fairness reputation, where $F(1, 120) = 146.62$, $p < .001$, $\eta_p^2 = .55$, 95% CI [.27, .47]. The post hoc analysis indicated that fair players ($M = 3.67 \pm .11$) were perceived as more altruistic than unfair players ($M = 1.91 \pm .12$). The main effect of role was significant, where $F(1, 120) = 15.62$, $p < .001$, $\eta_p^2 = .12$, 95% CI [.01, .13]. The post hoc analysis indicated that proposers ($M = 2.62 \pm .11$) were perceived as less altruistic than dictators ($M = 3.00 \pm .09$). The interaction between fairness reputation and role was significant, where $F(1, 120) = 31.23$, $p < .001$, $\eta_p^2 = .21$, 95% CI [.05, .19]. The simple effect analysis indicated that fair proposers ($M_{FP} = 3.28 \pm .13$) were perceived as more altruistic than unfair proposers ($M_{UP} = 1.96 \pm .14$), $p < .001$; fair dictators ($M_{FD} = 4.06 \pm .13$) were also perceived as more altruistic than unfair dictators ($M_{UD} = 1.86 \pm .12$), $p < .001$, with a larger difference than that for proposers. No other main effects or interactions were significant.

The ANOVA was conducted with cooperation ratings as the dependent variable. We found a significant main effect of fairness reputation, where $F(1, 120) = 445.67$, $p < .001$, $\eta_p^2 = .79$, 95% CI [.56, .71]. The post hoc analysis indicated that fair players ($M = 4.48 \pm .11$) were perceived as more cooperative than unfair players ($M = 1.91 \pm .07$). The main effect of role was significant, where $F(1, 120) = 31.33$, $p < .001$, $\eta_p^2 = .42$, 95% CI [.05, .19]. The post hoc analysis indicated

that proposers ($M = 3.12 \pm .09$) were perceived as less cooperative than dictators ($M = 3.45 \pm .09$). The interaction between role and SVO was significant, where $F(1, 120) = 5.29$, $p = .095$, $\eta_p^2 = .04$, 95% CI [.00, .07]. The simple effects tests indicated that for prosocial participants, proposers ($M = 3.08 \pm .11$) were perceived as less cooperative than dictators ($M = 3.34 \pm .10$), $p = .019$; and for individualistic participants, proposers ($M = 2.86 \pm .12$) were also perceived as less cooperative than dictators ($M = 3.49 \pm .11$), $p < .001$, with a larger difference than that for prosocial participants. No other main effects or interactions were significant.

The ANOVA was conducted with warmth rating as the dependent variable. We found a significant main effect of fairness reputation, with a Greenhouse-Geisser corrected $F(1, 120) = 721.36$, $p < .001$, $\eta_p^2 = .67$, $\varepsilon = 1.00$, 95% CI [.67, 1.00]. The post hoc analysis indicated that fair players ($M = 4.13 \pm .10$) were perceived as warmer than unfair players ($M = 1.94 \pm .06$). The main effect of role was significant, with a Greenhouse-Geisser corrected $F(1, 120) = 51.99$, $p < .001$, $\eta_p^2 = .34$, $\varepsilon = 1.00$, 95% CI [.13, 1.00]. The post hoc analysis indicated that proposers ($M = 2.74 \pm .07$) were perceived as less warm than dictators ($M = 3.33 \pm .08$). The interaction between fair reputation and role was significant, with a Greenhouse-Geisser corrected $F(1, 120) = 11.71$, $p < .001$, $\eta_p^2 = .03$, $\varepsilon = 1.00$, 95% CI [.01, 1.00]. The simple effects tests indicated that fair proposers ($M_{FP} = 3.70 \pm .11$) were perceived as warmer than unfair proposers ($M_{UP} = 1.78 \pm .06$), $p < .001$; fair dictators ($M_{FD} = 4.56 \pm .12$) were also perceived as warmer than unfair dictators ($M_{UD} = 2.09 \pm .08$), $p < .001$, with a larger difference than that for proposers. No other main effects or interactions were significant.

The ANOVA was conducted with competence ratings as the dependent variable. We found a significant main effect of fairness reputation, where $F(1, 120) = 9.75$, $p = .002$, $\eta_p^2 = .08$, 95% CI [.01, .10]. The post hoc analysis indicated that fair players ($M = 4.16 \pm .08$) were perceived as more competent than those unfair ($M = 3.79 \pm .11$). The main effect of role was also significant, where $F(1, 120) = 24.60$, $p < .001$, $\eta_p^2 = .17$, 95% CI [.03, .17]. The post hoc analysis indicated that proposers ($M = 3.77 \pm .09$) were perceived as less competent than dictators ($M = 4.18 \pm .09$). No other main effects or interactions were significant.

3.4 Homogeneity test of variance on social cognition of responders

For the ANOVA on fairness ratings, the Box's M test revealed significant differences in group covariance matrices, where Box's M = 10.46, $F(3, 42) = 3.42$, $p = .016$. For prosocial participants, the Levene's test on the variance of fairness ratings for responders was significant, where $F(1, 124) = 4.59$, $p = .028$, indicating that the variance of fairness ratings of fair responders ($S^2 = 2.44$) was significantly greater than that for unfair responders ($S^2 = 1.38$). For individualistic participants, the Levene's test on the variance of fairness ratings for responders was not significant, where $F(1, 116) = .96$, $p = .334$, indicating that there were no significant difference in the variance of fairness ratings between fair responders ($S^2 = 2.83$) and unfair responders ($S^2 = 2.20$) (Figure. S2A).

For the ANOVA on competence ratings, the Box's M test revealed significant differences in group covariance matrices, where Box's M = 8.50, $F(3, 42) = 2.78$, $p = .039$. For prosocial participants, the Levene's test on the variance of competence ratings for responders was not significant, where $F(1, 124) = 1.79$, $p = .181$, indicating that there were no significant difference in the variance of competence ratings between fair responders ($S^2 = 1.46$) and unfair responders ($S^2 = 1.12$). For individualistic participants, the Levene's test on the variance of competence ratings for responders was not significant, where $F(1, 116) = .47$, $p = .492$, indicating that there were no significant difference in the variance of competence ratings between fair responders ($S^2 = 1.76$) and unfair responders ($S^2 = 2.09$) (Figure. S2B).

Box's M test assessed no significant differences in group covariance matrices of other ratings (trustworthiness, altruism, cooperation or warmth).

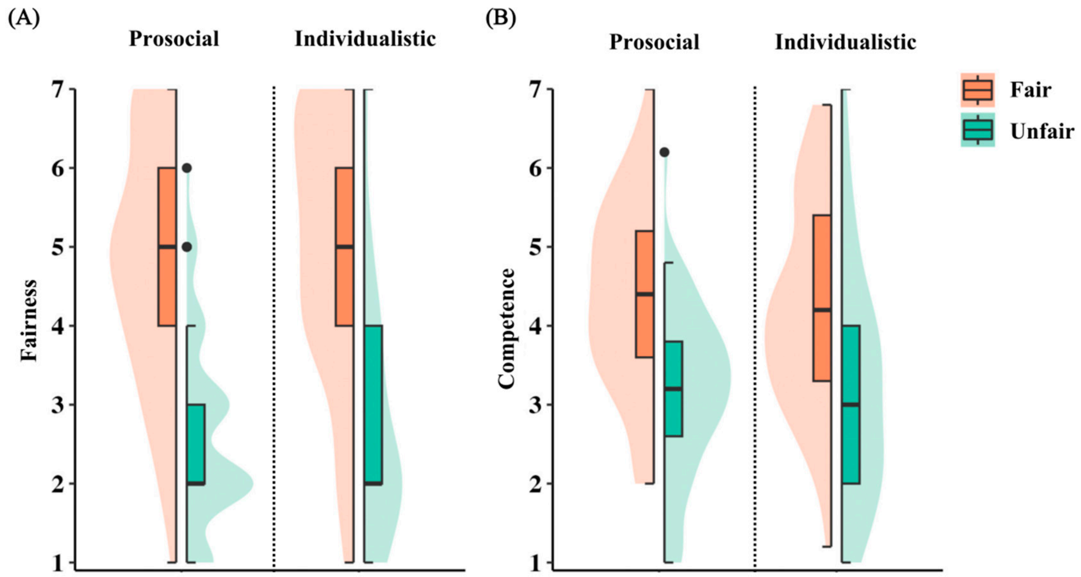

Figure S2. (A) For both prosocials and individualists, the variances of fairness ratings are greater for fair proposers and fair dictators with a wider distribution, than for unfair proposers and dictators with a narrower distribution. (B) Although Box's M test was significant, Levene's test found no significant differences in the variances of competence ratings between fair and unfair proposers or between fair and unfair dictators, for either prosocials or individualists.

3.5 ANOVA on social cognition of responders' fairness reputations

The ANOVA was conducted with fairness ratings as the dependent variable. We found a significant main effect of fairness reputation, where Greenhouse-corrected $F(1, 120) = 141.75$, $p < .001$, $\eta_p^2 = .53$, $\varepsilon = 1.00$, 95% CI [.44, 1.00]. The post hoc analysis indicated that fair responders ($M = 4.91 \pm .15$) were perceived as fairer than unfair responders ($M = 2.65 \pm .12$). No other main effects or interactions were significant.

The ANOVA was conducted with trustworthiness ratings as the dependent variable. We found a significant main effect of fairness reputation, where $F(1, 120) = 8.29$, $p = .005$, $\eta_p^2 = .07$, 95% CI [.00, .09]. The post hoc analysis indicated that fair responders ($M = 4.41 \pm .13$) were perceived as more trustworthy than unfair responders ($M = 3.90 \pm .15$). No other main effects or interactions were significant.

The ANOVA was conducted with altruism ratings as the dependent variable. We found a

significant main effect of fairness reputation, where $F(1, 120) = 194.77$, $p < .001$, $\eta_p^2 = .62$, 95% CI [.34, .53]. The post hoc analysis indicated that fair responders ($M = 2.78 \pm .13$) were perceived as less altruistic than unfair responders ($M = 5.37 \pm .15$). No other main effects or interactions were significant.

The ANOVA was conducted with cooperation ratings as the dependent variable. We found a significant main effect of fairness reputation, where $F(1, 120) = 48.56$, $p < .001$, $\eta_p^2 = .29$, 95% CI [.09, .25]. The post hoc analysis indicated that fair responders ($M = 3.79 \pm .14$) were perceived as less cooperative than unfair responders ($M = 5.09 \pm .15$). No other main effects or interactions were significant.

The ANOVA was conducted with warmth ratings as the dependent variable. We found a significant main effect of fairness reputation, where $F(1, 120) = 112.34$, $p < .001$, $\eta_p^2 = .48$, 95% CI [.22, .41]. The post hoc analysis indicated that fair responders ($M = 3.79 \pm .13$) were perceived as less warm than unfair responders ($M = 5.24 \pm .13$). No other main effects or interactions were significant.

The ANOVA was conducted with competence ratings as the dependent variable. We found a significant main effect of fairness reputation, where Greenhouse-corrected $F(1, 120) = 50.57$, $p < .001$, $\eta_p^2 = .30$, $\varepsilon = 1.00$, 95% CI [.19, 1.00]. The post hoc analysis indicated that fair responders ($M = 4.30 \pm .11$) were perceived as more competent than unfair responders ($M = 3.18 \pm .12$). No other main effects or interactions were significant.

4 Supplementary results of network analysis

4.1 Robustness Checks for Network Estimation

To ensure the robustness of the network estimation, we conducted supplementary analyses using both Pearson and polychoric correlations and varied the EBIC-GLASSO tuning parameter γ. After applying the nonparanormal (npn) transformation, Pearson correlations (corMethod = “npn”) were used in the main analysis. A robustness check with polychoric correlations, which are more suitable for ordinal Likert-type data, yielded highly similar network structures and centrality rankings (all Spearman correlations of centrality indices > 0.92).

For the EBIC-GLASSO tuning parameter, we compared networks estimated with γ = 0.25, 0.5 (main analysis), and 0.75. Spearman correlations of edge weights across γ values were extremely high: proposer (0.25 vs 0.5 = 0.983, 0.5 vs 0.75 = 1.000, 0.25 vs 0.75 = 0.983); responder (0.979, 1.000, 0.979); dictator (1.000, 1.000, 1.000). Centrality indices (Strength, Closeness, Betweenness, Expected Influence) were also highly consistent (all ≥ 0.885).

These results demonstrate that the network edge structures and centrality rankings are robust across correlation types and EBIC γ parameters.

4.2 Stability and accuracy of the social cognitive networks

The correlation stability (CS) coefficient was used to evaluate the centrality stability (Epskamp et al., 2018). The CS coefficients of strength, closeness, and EI centrality showed a qualified stability for all three networks, all above the recommended minimum threshold of >0.25. We interpreted our findings based on the above data. The results of centrality indices stability, edge-weight accuracy, and the bootstrapped difference tests for node centrality and edge weights were shown in Figure S3–5.

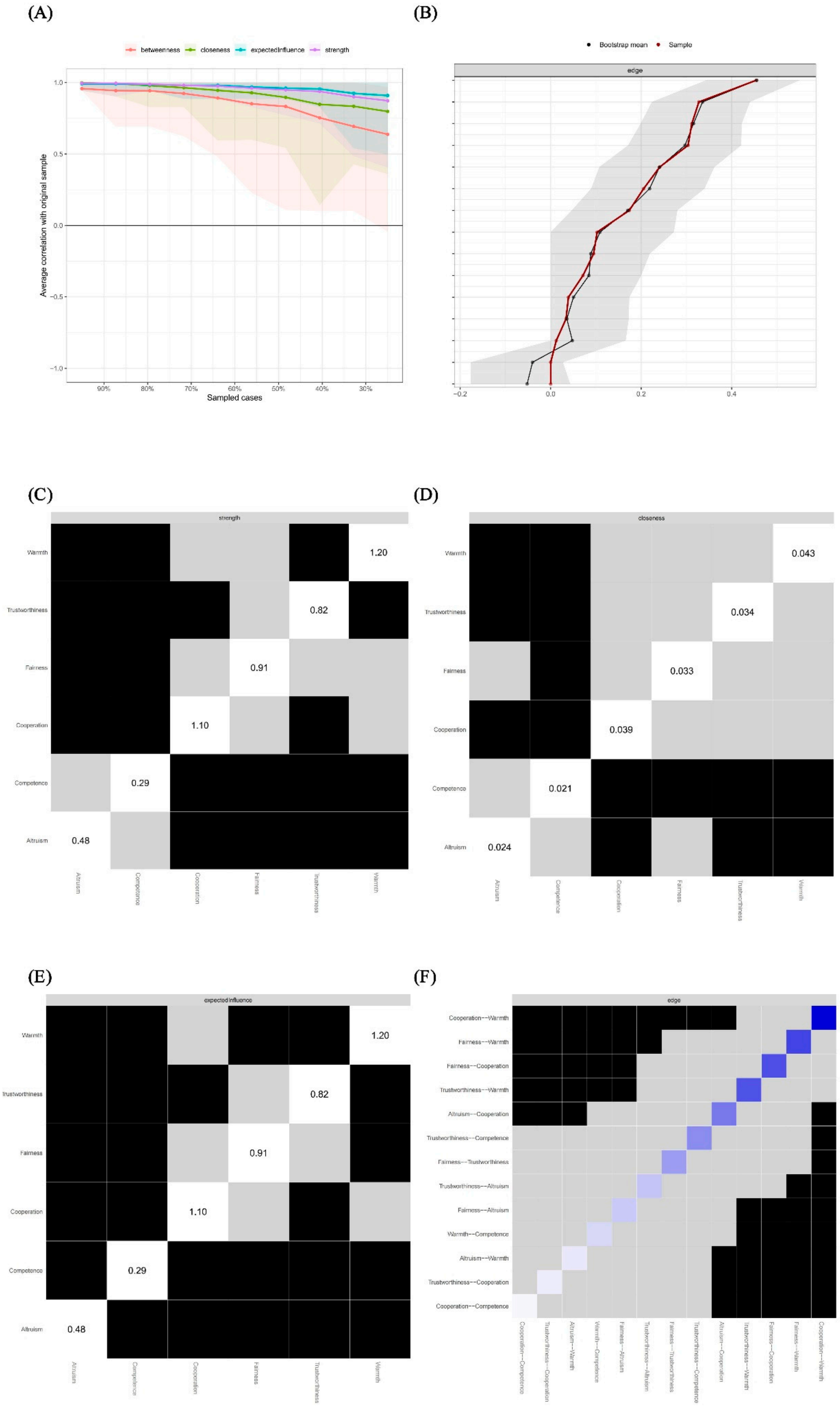

(A)
betweenness
closeness
expectedInfluence
strength
Average correlation with original sample
Sampled cases
1.0
0.5
0.0
−0.5
−1.0
90%
80%
70%
60%
50%
40%
30%
(B)
Bootstrap mean
Sample
edge
−0.2
0.0
0.2
0.4
(C)
strength
Warmth
Trustworthiness
Fairness
Cooperation
Competence
Altruism
1.20
0.82
0.91
1.10
0.29
0.48
(D)
closeness
Warmth
Trustworthiness
Fairness
Cooperation
Competence
Altruism
0.043
0.034
0.033
0.039
0.021
0.024
(E)
expected influence
Warmth
Trustworthiness
Fairness
Cooperation
Competence
Altruism
1.20
0.82
0.91
1.10
0.29
0.48
(F)
edge
Cooperation--Warmth
Fairness--Warmth
Fairness--Cooperation
Trustworthiness--Warmth
Altruism--Cooperation
Trustworthiness--Competence
Fairness--Trustworthiness
Trustworthiness--Altruism
Fairness--Altruism
Warmth--Competence
Altruism--Warmth
Trustworthiness--Cooperation
Cooperation--Competence

Figure S3. (A) Average correlation between centrality indices of the original whole sample and those estimated in subgroups obtained by dropping increasing percentages of subjects for the social cognition network of proposer. The lines indicate the means and the areas indicate the range from 2.5% to 97.5% quantile; (B) Bootstrapped confidence intervals of estimated edge-weights for the social cognition network of proposer. The red line indicates the values of edge-weight estimated in the whole sample, the grey area surrounding the red line represents the 95% quantile of the bootstrapped sampling distribution, and each horizontal line represents an edge of the network, ordered from highest to lowest edge-weight; (C) Bootstrapped difference test for node Strength centrality in the social cognition network of proposer. Black boxes indicate strengths that are significantly different from one another, and grey boxes indicate strengths that are not significantly different; (D) Bootstrapped difference test for node Closeness centrality in the social cognition network of proposer. Black boxes indicate closenesses that are significantly different from one another, and grey boxes indicate closenesses that are not significantly different; (E) Bootstrapped difference test for node Expected Influence in the social cognition network of proposer. Black boxes indicate Expected Influence (EI) that are significantly different from one another, and grey boxes indicate Expected Influence (EI) that are not significantly different; (F) Bootstrapped difference tests between edge-weights in the social cognition network of proposer. Black boxes indicate edges that were significantly different from one another. Grey boxes indicate edges that were not significantly different.

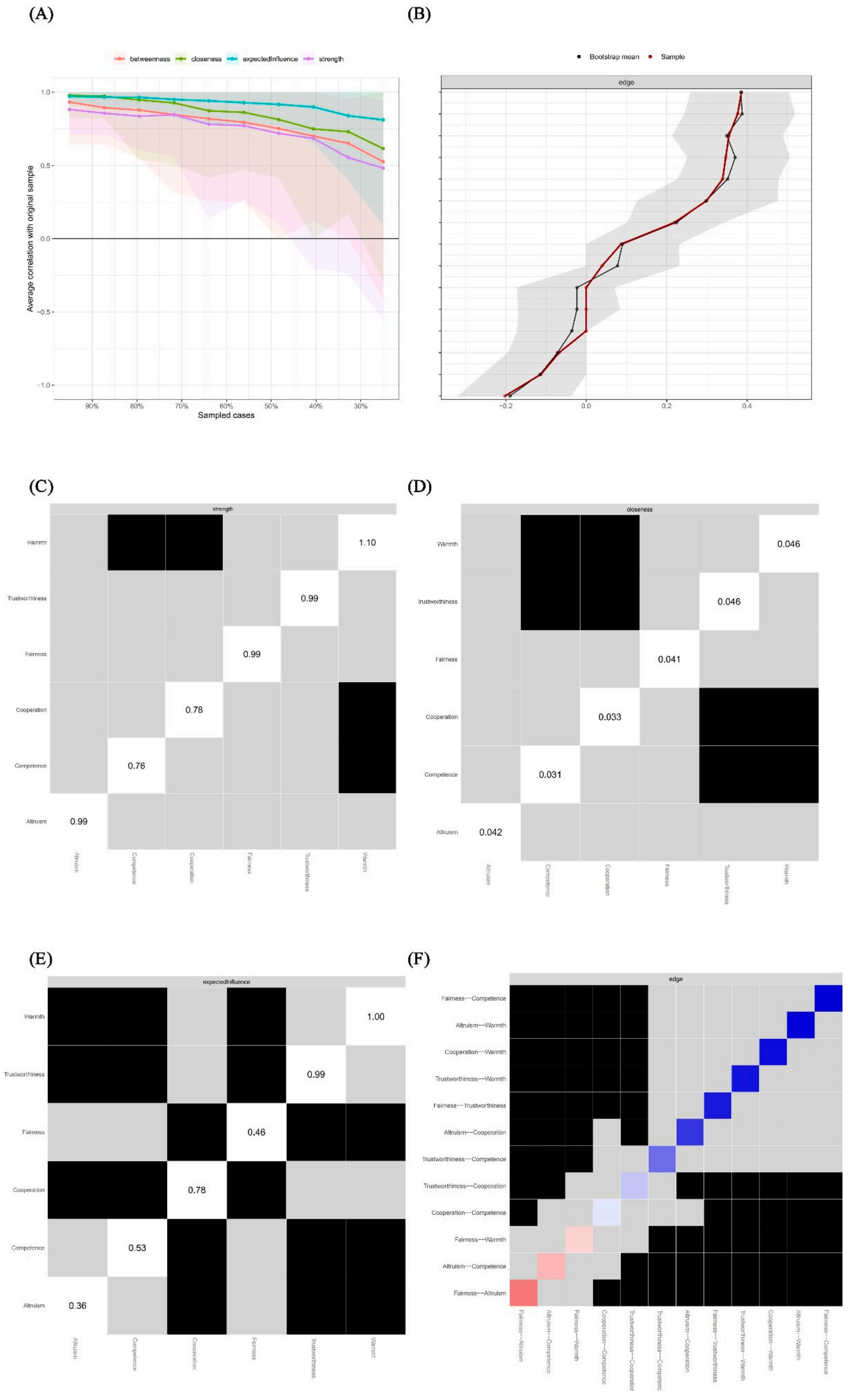

(A)
betweenness
closeness
expectedInfluence
strength
Average correlation with original sample
Sampled cases
90%
80%
70%
60%
50%
40%
30%
(B)
Bootstrap mean
Sample
edge
(C)
strength
Warmth
Trustworthiness
Fairness
Cooperation
Competence
Altruism
1.10
0.99
0.99
0.78
0.76
0.99
(D)
closeness
0.046
0.046
0.041
0.033
0.031
0.042
(E)
expectedInfluence
1.00
0.99
0.46
0.78
0.53
0.36
(F)
edge
Fairness--Competence
Altruism--Warmth
Cooperation--Warmth
Trustworthiness--Warmth
Fairness--Trustworthiness
Altruism--Cooperation
Trustworthiness--Competence
Trustworthiness--Cooperation
Cooperation--Competence
Fairness--Warmth
Altruism--Competence
Fairness--Altruism

Figure S4. (A) Average correlation between centrality indices of the original whole sample and those estimated in subgroups obtained by dropping increasing percentages of subjects for the social cognition network of responder. The lines indicate the means and the areas indicate the range from 2.5% to 97.5% quantile; (B) Bootstrapped confidence intervals of estimated edge-weights for the social cognition network of responder. The red line indicates the values of edge-weight estimated in the whole sample, the grey area surrounding the red line represents the 95% quantile of the bootstrapped sampling distribution, and each horizontal line represents an edge of the network, ordered from highest to lowest edge-weight; (C) Bootstrapped difference test for node Strength centrality in the social cognition network of responder. Black boxes indicate strengths that are significantly different from one another, and grey boxes indicate strengths that are not significantly different; (D) Bootstrapped difference test for node Closeness centrality in the social cognition network of responder. Black boxes indicate closenesses that are significantly different from one another, and grey boxes indicate closenesses that are not significantly different; (E) Bootstrapped difference test for node Expected Influence in the social cognition network of responder. Black boxes indicate Expected Influence (EI) that are significantly different from one another, and grey boxes indicate Expected Influence (EI) that are not significantly different; (F) Bootstrapped difference tests between edge-weights in the social cognition network of responder. Black boxes indicate edges that were significantly different from one another. Grey boxes indicate edges that were not significantly different.

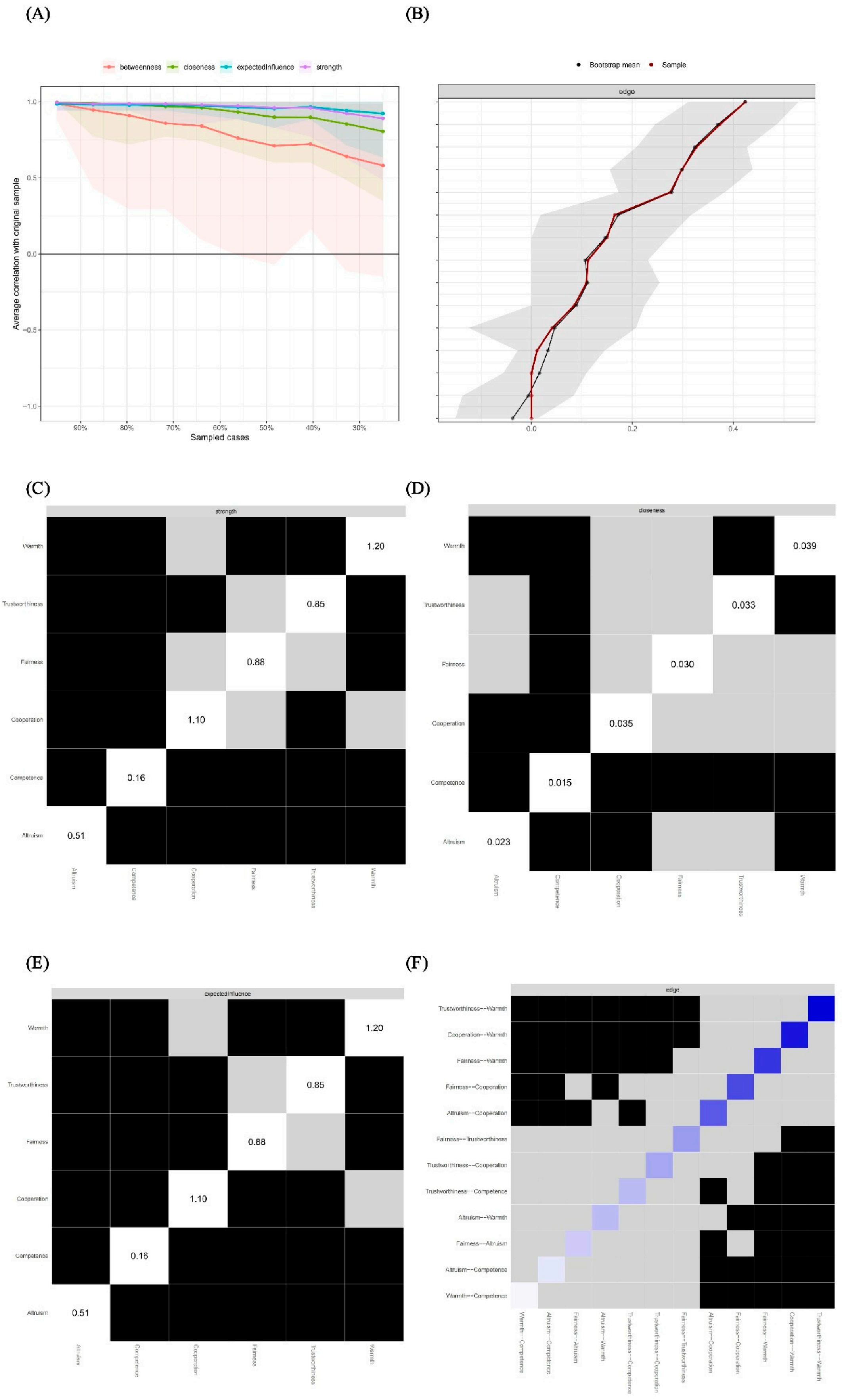
(A)
betweenness
closeness
expectedInfluence
strength
Average correlation with original sample
Sampled cases
1.0
0.5
0.0
−0.5
−1.0
90%
80%
70%
60%
50%
40%
30%
(B)
Bootstrap mean
Sample
edge
0.0
0.2
0.4
(C)
strength
Warmth
Trustworthiness
Fairness
Cooperation
Competence
Altruism
1.20
0.85
0.88
1.10
0.16
0.51
(D)
closeness
Warmth
Trustworthiness
Fairness
Cooperation
Competence
Altruism
0.039
0.033
0.030
0.035
0.015
0.023
(E)
expectedInfluence
Warmth
Trustworthiness
Fairness
Cooperation
Competence
Altruism
1.20
0.85
0.88
1.10
0.16
0.51
(F)
edge
Trustworthiness--Warmth
Cooperation--Warmth
Fairness--Warmth
Fairness--Cooperation
Altruism--Cooperation
Fairness--Trustworthiness
Trustworthiness--Cooperation
Trustworthiness--Competence
Altruism--Warmth
Fairness--Altruism
Altruism--Competence
Warmth--Competence

Figure S5. (A) Average correlation between centrality indices of the original whole sample and those estimated in subgroups obtained by dropping increasing percentages of subjects for the social cognition network of dictator. The lines indicate the means and the areas indicate the range from 2.5% to 97.5% quantile; (B) Bootstrapped confidence intervals of estimated edge-weights for the social cognition network of dictator. The red line indicates the values of edge-weight estimated in the whole sample, the grey area surrounding the red line represents the 95% quantile of the bootstrapped sampling distribution, and each horizontal line represents an edge of the network, ordered from highest to lowest edge-weight; (C) Bootstrapped difference test for node Strength centrality in the social cognition network of dictator. Black boxes indicate strengths that are significantly different from one another, and grey boxes indicate strengths that are not significantly different; (D) Bootstrapped difference test for node Closeness centrality in the social cognition network of dictator. Black boxes indicate closenesses that are significantly different from one another, and grey boxes indicate closenesses that are not significantly different; (E) Bootstrapped difference test for node Expected Influence in the social cognition network of dictator. Black boxes indicate Expected Influence (EI) that are significantly different from one another, and grey boxes indicate Expected Influence (EI) that are not significantly different; (F) Bootstrapped difference tests between edge-weights in the social cognition network of dictator. Bootstrapped difference tests between edge-weights in the social cognition network of dictator.

4.2 Centrality of social cognitive networks for the three roles

Table S1. The results from centrality of social cognitive networks

		Strength	Closeness	Betweenness	EI
	Fairness	.34	.10	-.91	.34
	Trustworthiness	.08	.22	.91	.08
Proposer	Altruism	-.91	-.99	-.91	-.91
	Cooperation	.76	.78	.91	.76
	Warmth	1.17	1.24	.91	1.17
	Competence	-1.45	-1.35	-.91	-1.45
	Fairness	-.35	.13	-.24	-.83
	Trustworthiness	.33	.99	1.22	1.09
Responder	Altruism	.32	.29	-.24	-1.17
	Cooperation	-1.11	-1.05	-.98	.33
	Warmth	1.37	.99	1.22	1.15
	Competence	-1.26	-1.35	-.98	-.56

Dictator	Fairness	.22	.11	-.88	.22
	Trustworthiness	.15	.43	.63	.15
	Altruism	-.71	-.70	-.88	-.71
	Cooperation	.77	.66	.63	.77
	Warmth	1.15	1.12	1.38	1.15
	Competence	-1.58	-1.62	-.88	-1.58

Note: EI: expected influence.

4.3 Differences in edge-weights of the Social cognitive networks of three roles

Table S2. The results from differences in edge-weights of the social cognitive networks of proposer, responder and dictator.

Edges	Edge-weights			*p* value [a]	*p* value [b]	*p* value [c]
	Proposer	Responder	Dictator			
Warmth-Fairness	.33	-.07	.33	.015	.015	1
Warmth-Trustworthiness	.30	.35	.42	1	1	1
Warmth-Cooperation	.45	.35	.37	1	1	1
Warmth-Altruism	.04	.38	.11	.044	.264	1
Warmth-Competence	.07	0	0	1	1	1
Competence-Fairness	0	.39	0	.015	.015	1
Competence-	.21	.22	.11	1	1	1

Trustworthiness						
Competence-Cooperation	.01	.04	0	1	1	1
Competence-Altruism	0	-.11	.04	1	1	1
Fairness-Trustworthiness	.17	.34	.17	.749	.559	1
Fairness-Cooperation	.31	0	.30	.015	.015	1
Fairness-Altruism	.09	-.20	.09	.015	.015	1
Trustworthiness-Cooperation	.03	.09	.15	1	1	1
Trustworthiness-Altruism	.10	0	0	.809	1	1
Cooperation-Altruism	.24	.30	.28	1	1	1

Note: The Network Comparison Test (NCT), a two-tailed permutation test, was conducted to examine the differences between the edge-weights of two networks. P value a indicated significant level of the edge-weight differences between proposer and responder after Holm-Bonferroni correction. P value b indicated significant level of the edge-weight differences between responder and dictator after Holm-Bonferroni correction. P value c indicated significant level of the edge-weight differences between proposer and dictator after the Holm-Bonferroni correction.

4.4 Exploratory Network Comparison Based on SVO

We compared the social cognitive networks between prosocial and individualistic participants using the Network Comparison Test (NCT). For proposers, no significant group differences were observed in either global network strength (strength difference = .002, $p = .580$) or overall structure (maximum edge weight difference = .04, $p = .267$). For responders, both global strength (strength difference < .001, $p = .845$) and structure (maximum edge weight difference = .03, $p = .168$) were nonsignificant, except for one edge (warmth – cooperativeness) showing a significant difference ($p < .05$), indicating a stronger warmth-cooperativeness connection in the prosocial group. For dictators, no significant group differences were found (strength difference < .001, $p = .595$; maximum edge weight difference < .001, $p = .962$). The results of exploratory network comparisons between prosocial and individualistic participants for the proposer, responder, and dictator are shown in Figure S6.

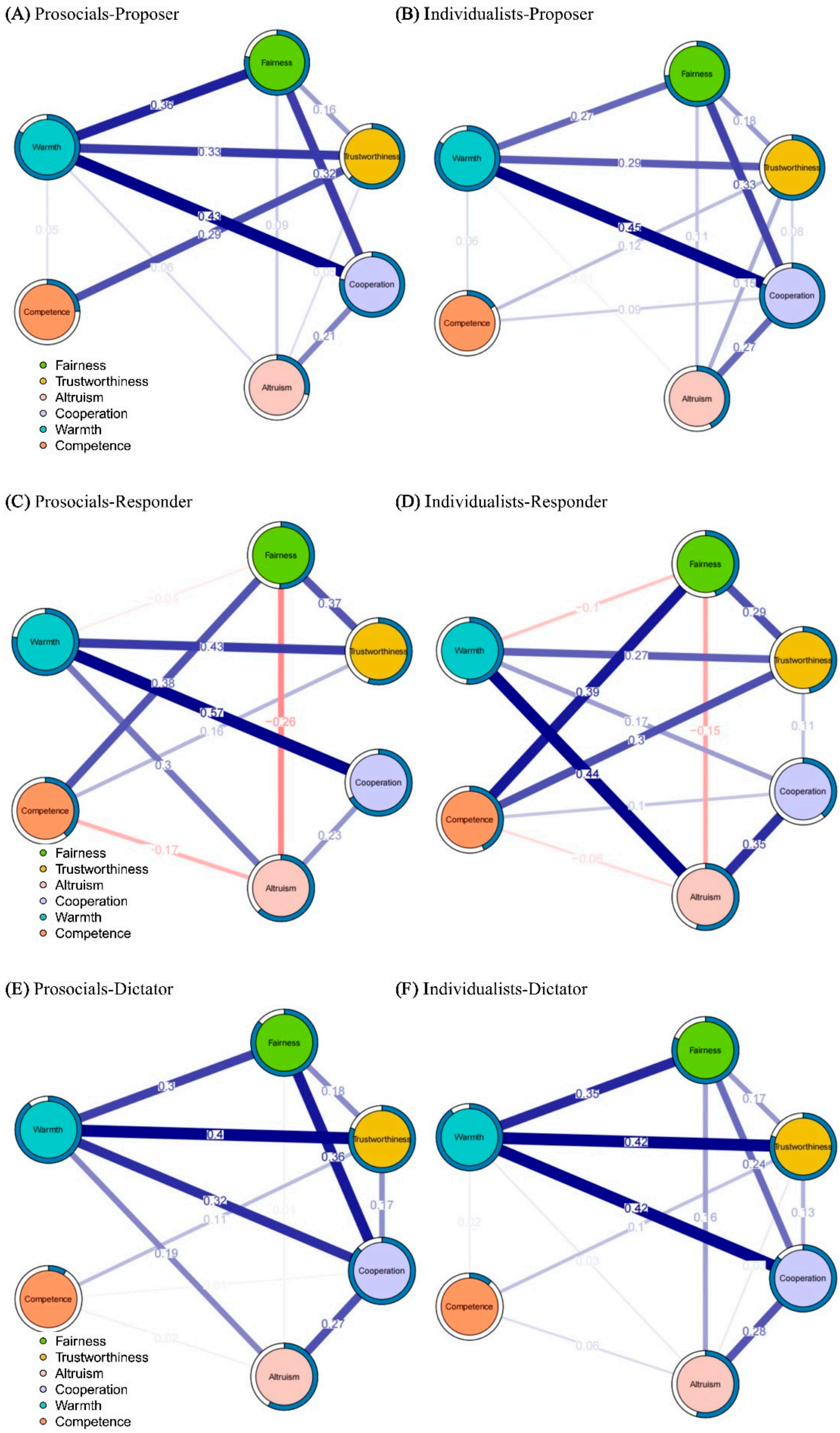

Figure S6. The results of exploratory network comparisons between prosocial and individu alistic participants for the proposer, responder, and dictator.

Reference

Epskamp, S., Borsboom, D., & Fried, E. I. (2018). Estimating psychological networks and their accuracy: A tutorial paper. *Behavior research methods*, *50*, 195-212. https://doi.org/10.3758/s13428-017-0862-1

Murphy, R. O., Ackermann, K. A., & Handgraaf, M. J. (2011). Measuring social value orientation. *Judgment and Decision making*, *6*(8), 771-781. https://doi.org/10.1017/S1930297500004204
